# Clinical experience can compensate for inferior academic achievements in an undergraduate objective structured clinical examination

**DOI:** 10.1186/s12909-023-04082-x

**Published:** 2023-03-16

**Authors:** Stefanos A. Tsikas, Kambiz Afshar

**Affiliations:** 1 Academic Controlling, Hannover Medical School, D-30625 Hannover, Carl-Neuberg-Str. 1 Germany; 2 Institute for General Practice and Palliative Care, Hannover Medical School, Hannover, Germany

**Keywords:** Academic success, Student selection, Medical education, Medical students

## Abstract

**Background:**

Practical and non-cognitive skills are essential to medical professions; yet, success in medical studies is primarily assessed with cognitive criteria. We show that practical exams can benefit students who have only average high school final grades, but working experience in medical professions.

**Methods:**

With a cross-sectional study, we compare the performance of undergraduate medical students with working experience in adjacent health-care professions (and below-average school leaving-grades) with students who entered medical school directly based on their excellent school records in an Objective Structured Clinical Examination (OSCE). For a sample of more than 1,200 students, we use information on OSCE scores in medical and practical skills, doctor-patient communication/interaction, performance in MC-exams, and core sociodemographic variables.

**Results:**

Waiting list students outperformed their classmates in the demonstration of practical skills. Students admitted via their excellent school grades scored best overall. This difference vanishes once we control for school-leaving grade and age, the two main factors separating the analysed groups. Students from the waiting list have a significantly smaller overall chance to reach excellent grades in the first two years of study.

**Conclusions:**

Students who gathered experiences in health-care professions before enrolling at medical school can benefit from an expanded role of practical elements in medical studies. Student selection instruments should take these different starting positions and qualities of applicants into account, for example with a quota for the professionally experienced.

**Supplementary Information:**

The online version contains supplementary material available at 10.1186/s12909-023-04082-x.

## Introduction

### Background

Admission to German medical schools predominantly relies on cognitive criteria, i.e. school-leaving grades (the German Abitur, comparable to A-levels in the UK or the Grade Point Average (GPA) in the US) and study ability tests. These selection criteria have a sound predictive validity for study success at medical school. Non-cognitive selection criteria such as interviews or personality assessments, on the other hand, are unrelated to academic outcomes (see the excellent reviews of the relevant literature by Deary et al. [1], Patterson et al. [2], and Meyer et al. [3]). This finding is, however, unsurprising, because performance assessments are mostly multiple choice (MC) tests that target and favour cognitive abilities. A more suitable assessment tool in competency-based medical education is the ‘Objective Structured Clinical Examination’, which simulates practitioners’ job routines and specifically targets practical, clinical and communicative skills.

Even if they are under-represented in exams, traits targeted with non-cognitive selection criteria (practical experiences, empathy, responsibility, confidence in patient-doctor communications and interactions) are undoubtedly crucial soft-skills required in the medical profession [4, 5]. In recent years, several educational policy reforms in Germany have sought to strengthen the role of practical and social skills in medical curricula, which resulted inter alia in the passage of a national competency-based learning objective catalogue (‘Nationaler Kompetenzbasierter Lernzielkatalog’ (NKLM) [6, 7]) and in drafts for an updated medical licensure act.

The ongoing demographic change with a looming shortage of physicians represents further challenges for the German health care system and arguably justifies a need for more medical students. Especially care provided by general practitioners in rural areas is compromised. Some German states have implemented a special quota for applicants who pledge to become general practitioners in rural areas. More broadly, the aim is to attract students with an affinity to primary care, implying for example a higher relevance of practical skills already in the selection process.

Up to and including the year 2019, students who received vocational training and often worked as e.g. paramedics, trained nurses or medical technicians, were admitted via the so-called waiting-list quota, which allowed applicants to eventually study medicine even though their Abitur grades were not good enough to be considered in other selection quotas (see the section ‘Methods’). Interestingly, actually receiving vocational training was neither required for admission nor increased applicants’ chances to jump up spots on the waiting list. Nevertheless, virtually all persons enrolled via the waiting list have experiences in relevant health-care professions, speaking for their high intrinsic motivation and determination to pursue careers as physicians. In recent years, the average waiting time on the list was substantial, at Hannover Medical School (MHH), for which we report our results, on average more than five years before a person was eventually offered a place. Therefore, students from the waiting list significantly differed from their classmates not only in their Abitur grade (or cognitive aptitude), but also in their age at admission to medical school.

Because of the increasingly sprawling waiting time and a court ruling calling for changes in universities’ selection processes, in 2020 the waiting list arrangement was replaced by an eligibility quota for applicants who attest experience in selected health-care professions and performed well in a study ability test. Additionally, vocational training can be a bonus in selection procedures using school-leaving grades and performances in study ability tests. In our study, students joined medical school no later than 2019, thus, the waiting-list rule was still in place.

### Related literature

Literature on the link between clinical experience and (other) student selection instruments on study success is relatively scarce, although prior professional experiences might be connected to educational outcomes: students who have worked in health care before could have advantages in contextualizing theoretical knowledge [8]. Having experienced stress in the workplace could facilitate stress resilience within the study curriculum [9]. Chisholm-Burns et al. [10] concluded that higher perceived stress levels are associated with worse academic performance. For two German medical schools, Amelung et al. [9] found that vocational training has some predictive validity for study success in all exams during the first two years of study. Unfortunately, the authors did not analyse whether this finding was driven by e.g. oral or practical exams. For our sample of students, we will make this distinction in the course of our analyses.

The OSCE with its emphasis on clinical practice is an exam type where one could expect positive effects of working experience on study success. Martin et al. [11] found that performance in the examination relates to well-organized study methods, but not to clinical experience. However, the authors suggest that knowledge from practical work might relate to specific learning approaches. Chan et al. [12] tested if experiences in public speaking or performances (music, arts, drama) affected the results of an OSCE examination and found modest positive effects. Matet et al. [13] showed for a French medical school that OSCE results in behaviour-oriented stations (weakly) correlate with higher traineeship skills, while competence-oriented stations had only ties with performances in MC-exams. Kirton and Kravitz [14] concluded that OSCEs and MC-exams measure different competencies by explicitly targeting clinical skills.

In student selection procedures, interviews seek to capture non-cognitive and interpersonal skills. Studying ties between interview and OSCE performance, Basco et al. [15] and Tsikas [16] do not find any statistical evidence for such links. Generally, literature reviews and meta-studies find that very few selection methods other than school GPAs or cognitive aptitude tests predict study success [2, 17, 18].  Only Multiple-Mini Interviews (MMI), which mimic the OSCE’s structure, have shown predictive validity for study success in practical assessments [19–21].

### Research objectives

In our article, we compare study success of waiting list students with those students that were admitted based on their excellent Abitur grade. We consider all written and oral exam performances during the first two years of study, but focus on the OSCE, and specifically on the parts assessing medical and communicative skills. Because students who entered medical school directly after their graduation from High School lack practical experience, waiting list students should have a head start in practice-oriented parts of the OSCE, despite considerably poorer Abitur grades. Summarizing, we address the following research questions:

1. Do students with clinical experience outperform their classmates (who entered medical school based on excellent school leaving grades) in the demonstration of medical and communicative skills?
2. Are high cognitive abilities (i.e. the Abitur grade) positively associated with more competence- and communication oriented parts of the OSCE?
3. How are OSCE results (particularly in skills-based stations) related to overall academic success in the first two years of study?

## Methods

### Study design

We analyse the observed OSCE performance of more than 1,200 medical students who took the examination between 2015 and 2020 at the end of the second years of studies. In these years, the student selection process at MHH used four different quotas: in a first step, applicants from specific groups (e.g. hardship cases, prospective military doctors, or international students) were admitted upfront via special quotas. Of the remaining places, 20% went to those applicants with the best Abitur grades overall (*numerus clausus* (NC) quota). A majority of 60% of places was allocated with a mixture of Abitur grade and an interview with a selection committee (selection quota) [22]. The remaining 20% of places were reserved for applicants on the waiting list. Apart from the special quotas, placement in the different quotas depended almost entirely on the school leaving grade, the German ‘Abitur’. Passing grades are in the range from 1.0 (best) to 4.0 (worst passing grade) in steps of 0.1 points, and applicants in the NC and selection quota needed an excellent Abitur (1.0–1.4, corresponding to an ‘A’) to be admitted to medical school. After several years on the waiting list, also applicants with an Abitur grade of 2.7 or higher were admitted, corresponding to a B- or C.

**Fig. 1:**
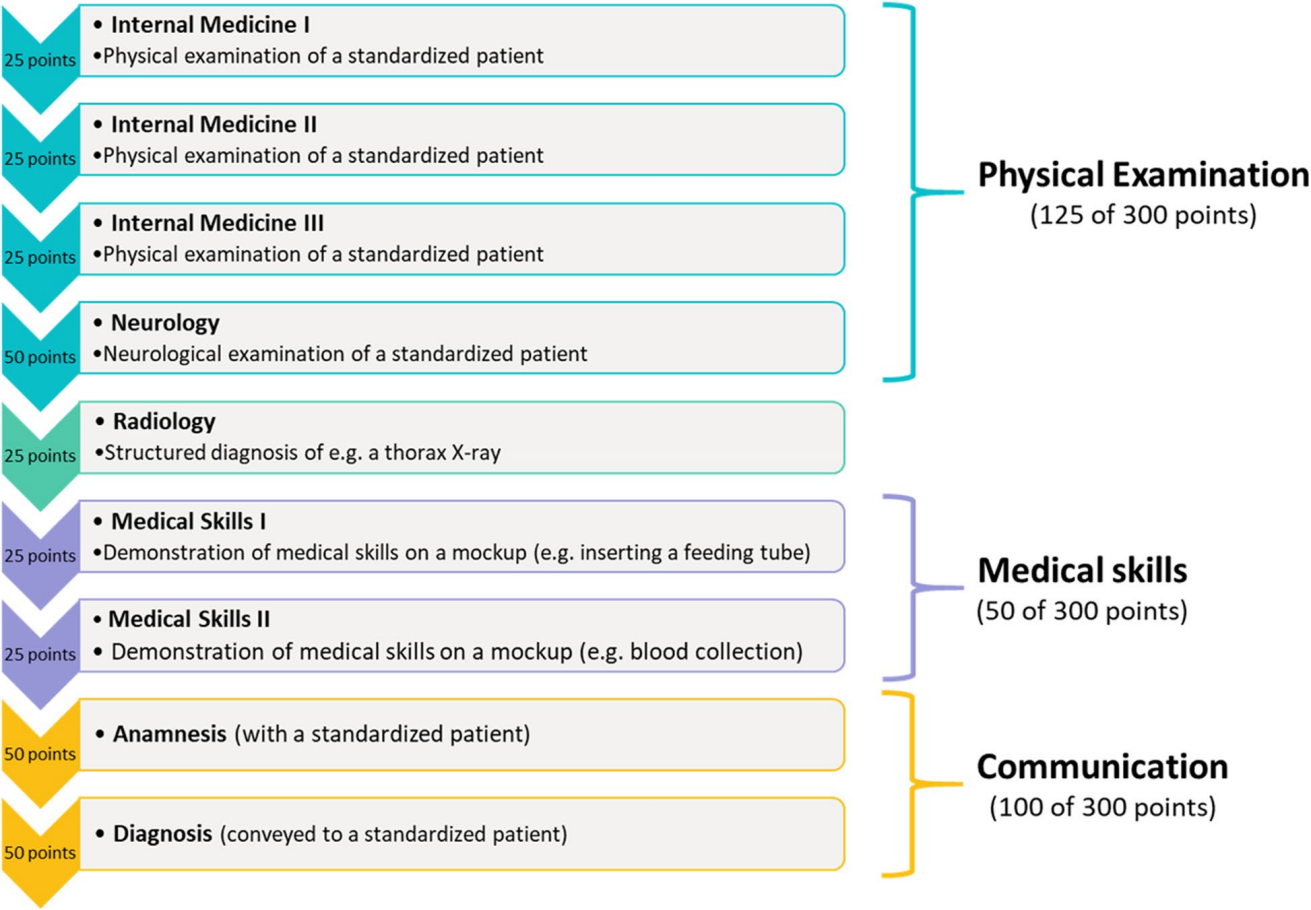
The OSCE at Hannover Medical School Note: Stations worth 25 points last 7 min, 50 points can be achieved in 15 min

Available to us were detailed exam results in all parts of the OSCE; we use the percentage of the maximum achievable points (per station and overall, see Fig. 1) in the OSCE as outcome variables. Our main variable of interest is a binary indicator denoting the admission quota of respective students. Sociodemographic control variables are students’ age, gender, nationality, and educational background. Furthermore, we can control for Abitur grades and students’ exam results (mainly in MC format) during their first two years of study. Lastly, we use cohort fixed effects to control for potential differences in the (sociodemographic) composition of student cohorts over time.

In the empirical analysis, we, first, perform non-parametric sample tests in order to analyse differences in students’ performance in the OSCE segments, with a special focus on practical and communicative skills. We prefer non-parametric tests to linear sample tests because exam grades, age etc. do not follow a normal distribution in our sample of students. In a next step, we perform linear regression analyses to control for the potential impact of sociodemographic characteristics and Abitur grade on the observed differences between the selection quotas. With the regression analysis, we can quantify performance differences of waiting list or selection students to the NC group, while taking into account Abitur grades, other exams and sociodemographic characteristics that might influence the OSCE results. Finally, we use an ordered logistic regressions to analyse how OSCE and overall success in M1 relate and discern between the NC, selection, waiting list and special quotas. Thus, we estimate and graphically illustrate the expected probabilities to reach, for example, a very good or just a satisfactory grade in the OSCE, given the parameters and variables discussed in this section.

### Setting

The MHH OSCE takes place at the end of the second academic year and is the final examination in the module ‘Diagnostic Methods’. In Hannover, the OSCE is set as the last examination en route to the first part of the German Medical Licensing Exam (M1). In the M1-phase, teaching mainly evolves around pre-clinical groundwork in physiology, anatomy, chemistry, psychology and diagnostic methods. At MHH, M1 is obtained with more than 10 graded written and oral (including the OSCE) exams. The final grade is the average of these exam results and called ‘M1-equivalence’, because at most German medical schools M1 is held as one large written and one oral exam after two years of study. Passing grades range from 1 (best) to 4 (worst) in steps of 1, equivalent to an A-D grading system. For simplicity, we use ‘M1’ instead of the term ‘M1-equivalence’ in this paper. Students at MHH do not need to have passed all preceding exams to participate in the OSCE, and can start taking courses in the third year without having passed the structured clinical examination or all other exams in the M1-phase.

The OSCE at MHH is a course of nine stations. Within ‘Physical examination’, students have to demonstrate their ability to perform a check-up in internal medicine and neurology on a standardised patient (e.g. of the heart, lungs, abdomen, and the nervous system); Fig. 1 shows that students can earn a total of 125 points in this part. Additionally, a structured diagnosis of an X-ray image is worth up to 25 points. Dettmer et al. [23] give a detailed description of the OSCE part ‘Radiology’ at MHH. In two further stations, students have to demonstrate their practical skills, for example by collecting blood or by performing an intra-muscular injection on a model and can earn 50 points. Lastly, up to 100 points are assigned in a medical consultation by conducting an anamnesis and conveying a diagnosis to a standardised patient (see also von Lengerke et al. [24]).

Stations worth 25 points have to be completed in seven minutes, stations worth 50 points in 15 min. Overall, the course lasts 95 min, including a one-minute changeover time between stations. Sixty percent of points have to be reached to pass the exam, 70% or more are equal to the grade ‘3’ (corresponding to a ‘C’), 80% or more for a ‘good’ 2 (B), and students who reached more than 90% are rewarded with the best grade “1” (A). Achieving less than 60% in one or several stations does not automatically result in a failed exam, because underperformance can be compensated with good results in other parts of the OSCE.

## Results

### Descriptive sample statistics

Table 1 shows that 63% of our sample are females. Women were particularly successful in the selection process, while the proportion is lowest in the special quotas. The average age at the time of enrolment was 21 years. The NC and selection groups, who predominantly begin their studies shortly after leaving school, are slightly younger. The increasingly long waiting period is evident in the age of the waiting list students, which is 29 years and thus almost ten years above NC.

**Table 1:** Sociodemographic sample statistics

	**Total**	**NC**	**Waiting list**	**Selection**	**Special quotas**
Female	63.38%	60.99%	61.05%	68.18%	55.31%
German	91.50%	98.90%	95.79%	95.18%	70.97%
Gymnasium	77.47%	82.97%	71.05%	82.77%	62.39%
Age	21.34 (4.07)	19.44 (1.95)	28.71 (4.12)	19.78 (1.85)	21.4 (3.23)
Abitur grade	1.53 (0.59)	1.01 (0.03)	2.62 (0.48)	1.31 (0.15)	1.69 (0.56)
*N* [%]	1283	182 [14.19%]	190 [14.81%]	685 [53.39%]	226 [17.61%]

Standard deviations are in parentheses. Age: mean age at enrollment. Average Abitur grades per quota are shown; *NC* Numerus clausus

Well above 90% of students are German nationals. Only in the special quotas (71%) the share is markedly lower, because international students are sorted here. Slightly more than 77% of students obtained their Abitur at a regular Gymnasium (academic High School); the share is highest in the NC group. Waiting list students visited other school forms such as integrated schools or night schools comparably more often. Table 1 further shows that NC students and the selection group had to have an almost perfect school-leaving grade (1.0) to enter medical school. The Abitur grade in the waiting list is 2.6 – at best average in a nation-wide comparison of High school graduates.

### Non-parametric sample tests

Table 2 reveals that, overall, students performed ‘good’ (80% or more of the point-total) in the OSCE. NC students perform best, and significantly better (*p* < 0.001, Mann–Whitney *U*-test) than all other groups. The OSCE result of the selection group is significantly above the waiting list students, who in turn are more successful than their classmates from the special quotas (*p* < 0.05).

**Table 2:** Outcome measures for study success

	**Total**	**NC**	**Waiting list**	**Selection**	**Special quotas**
OSCE (%)	82.9	85.63	81.34	83.88	78.98
	(7.14) [84]	(5.86) [86.67]	(7.39) [82.33]	(5.49) [84.33]	(10) [80.17]
M1 success (%)	90.81	95.58	80.11	94.86	83.56
M1 (overall)	2.04	1.67	2.31	1.98	2.34
	(0.66) [2]	(0.61) [2]	(0.67) [2]	(0.60) [2]	(0.65) [2]
M1 (written)	2.32	1.81	2.54	2.32	2.62
	(0.68) [2]	(0.64) [2]	(0.63) [3]	(0.63) [2]	(0.66) [3]
M1 (oral)	2.18	1.88	2.46	2.1	2.49
	(0.71) [2]	(0.71) [2]	(0.73) [2]	(0.65) [2]	(0.72) [2]
*N* [%]	1,283	182 [14.19%]	190 [14.81%]	685 [53.39%]	226 [17.61%]

Standard deviations are in parentheses, median levels are in square brackets. M1 (overall): all exams in the first two years of study. M1 (written): MC exams in the first two years of study. M1 (oral) includes the OSCE grade. M1 passing grades are 1, 2, 3,and 4. Abitur passing grades are in the range of 1.0–4.0, in steps of 0.1. M1 success: M1 obtained after passing the OSCE

*M1 success* takes the value 1 if all other exams mandatory for reaching M1 were passed before the OSCE (else it is 0). While this is the case for 95% of NC and selection students, 20% from the waiting list had not previously passed all other MC or oral exams in the first two years of study. Because most exams during M1 are MC tests, we include students’ final M1 grades in Table 2 as a means of comparison to the OSCE result: NC students beat all other groups, including selection students, by some distance. Waiting list and special quotas perform quite similarly, but significantly worse (*p* < 0.001, Mann–Whitney *U*-Test) than the selection group.

As Table 3 shows, NC and Selection students perform significantly better than waiting list and special quotas students in the OSCE part internal medicine (*p* < 0.001, Mann–Whitney *U*-test). The same holds for the neurological examination and the X-ray diagnosis, where the percentage-difference between NC, selection and waiting list students is even greater than in the physical examinations (see Fig. 1).

**Table 3:** OSCE scores (in %) per category

	**Total**	**NC**	**Waiting list**	**Selection**	**Special quotas**
Internal medicine	80.64	83.85	78.18	81.50	77.44
	(9.58) [81.33]	(8.94) [85.33]	(9.99) [78.67]	(8.55) [82.67]	(11.29) [78.67]
Neurology	78.17	80.93	76.61	79.18	74.96
	(13.64) [80]	(12.54) [84]	(14.63) [78]	(12.79) [82]	(15.25) [78]
Radiology	82.23	89.66	77.89	83.87	74.96
	(15.50) [84]	(11.12) [92]	(16.71) [80]	(13.86) [88]	(18.17) [80]
Medical skills	88.96	89.22	90.44	89.36	85.67
	(9.60) [92]	(8.71) [92]	(8.78) [92]	(9.13) [92]	(11.52) [88]
Communication	84.10	86.35	82.33	85.11	80.67
	(8.23) [86]	(6.93) [87]	(8.94) [85]	(7.11) [86]	(10.23) [82]

Standard deviations are in parentheses, median values are in square brackets. All mean values show percentages

While the share of scored points is on average 80% (thus, a ‘good’ grade by a whisker) in internal medicine, the assessment of medical skills (e.g. collecting blood, performing intramuscular injections or an electrocardiogram (ECG)) seems to be the ‘easiest’ part of the OSCE: students reach almost 90% of the possible 50 points. In absolute terms, the waiting list group is on top, but the difference to the NC and selection groups is narrow. Only students from the special quotas perform significantly worse (*p* < 0.001, Mann–Whitney *U*-test) than all other groups, but still acquire on average 86% of the 50 points at both medical skills stations.

In communication (anamnesis and imparting the diagnosis of an illness to a standardised patient), NC students are once more the most successful group, leaving their selection (*p* = 0.06) and waiting list (*p* < 0.001) peers behind them with a statistically significant difference (Mann–Whitney *U*-tests). However, the percentage-point gap between the top and bottom performers is slightly narrower than in the physical examinations.

### Student selection and OSCE success – regression analyses

#### Linear regression analysis

Columns (1)-(3) of Table 4 use the overall OSCE score as the dependent variable in our regression models. Column (1) of Table 4 largely reiterates the results of Table 2: compared with the NC group, all other students perform significantly worse in the OSCE, after controlling for potential cohort-specific differences with the addition of fixed effects. In specification (2), we include gender, nationality, and educational background as sociodemographic control variables: female students perform slightly (but statistically significantly) better than their male classmates (*p* < 0.05). Being German results in, on average, 5 percentage points more obtained in the OSCE, compared to internationals (i.e., non-Germans). Students who got their Abitur from a regular Gymnasium (i.e. academic High School) scored almost 2.5 percentage points more in the OSCE than those who visited other schools. Adding the covariates slightly alters the magnitudes of the selection-quota coefficients, but leaves statistical significance and overall picture unchanged.

**Table 4:** Student selection and OSCE performance, linear regressions

	(1)	(2)	(3)	(4)	(5)	(6)	(7)	(8)	(9)
	OSCE	OSCE	OSCE	OSCE	OSCE	OSCE	OSCE	OSCE	OSCE
Dep. Variable:	Total (%)	Total (%)	Total (%)	Skills (%)	Skills (%)	Skills (%)	Comm. (%)	Comm. (%)	Comm. (%)
**Quota**									
Waiting list	-4.305***	-3.835***	2.782**	0.364	0.681	6.689***	-4.032***	-3.621***	-1.672
	(0.685)	(0.664)	(1.299)	(0.890)	(0.893)	(1.920)	(0.831)	(0.818)	(1.663)
Selection	-1.687***	-1.564***	-0.635	-0.427	-0.364	0.468	-1.213**	-1.096*	-0.684
	(0.479)	(0.472)	(0.509)	(0.711)	(0.716)	(0.770)	(0.578)	(0.574)	(0.630)
Special quotas	-6.595***	-4.596***	-2.077**	-4.203***	-3.068***	-0.798	-5.589***	-3.536***	-2.550**
	(0.811)	(0.828)	(0.940)	(0.992)	(1.007)	(1.247)	(0.857)	(0.810)	(1.011)
Female		1.143***	1.124***		0.570	0.555		1.509***	1.465***
		(0.398)	(0.391)		(0.547)	(0.544)		(0.474)	(0.476)
German		5.301***	6.477***		2.682**	3.745***		5.771***	6.187***
		(0.916)	(0.947)		(1.263)	(1.280)		(1.182)	(1.239)
Gymnasium		2.435***	2.023***		1.862***	1.473**		1.868***	1.943***
		(0.510)	(0.511)		(0.722)	(0.725)		(0.601)	(0.635)
Age			-0.271***			-0.253**			0.018
			(0.089)			(0.120)			(0.112)
Abitur grade			-2.570***			-2.289**			-1.306
			(0.680)			(0.916)			(0.887)
Cohort-FE	*YES*	*YES*	*YES*	*YES*	*YES*	*YES*	*YES*	*YES*	*YES*
*N*	1272	1272	1272	1271	1271	1271	1271	1271	1271
*R*^2^	0.103	0.191	0.216	0.069	0.086	0.098	0.061	0.127	0.129

*Note*: Ordinary Least Squares (OLS) regressions. Robust standard errors are in parentheses. For admission quotas, coefficients report the percentage-point change relative to the NC quota

*p < 0.1

**p < 0.05

***p < 0.01

At the outset of this paper, we argued that students’ age and Abitur are the most obvious parameters that set the professionally experienced and the students with excellent school leaving grades apart. Indeed, one additional year at the start of studies is associated with 0.27 percentage points less in the OSCE, and a 0.1 (on a scale from 1.0 to 4.0) point inferior Abitur is connected to an ample 2.6 percentage points less in the exam. Adding age and Abitur grade has also a strong impact on the quota-coefficients in column (3) of Table 4. Students from the special quotas still have a significantly poorer outcome (although the disparity narrows, and the selection and NC groups are now inseparable), but waiting list students now score significantly better in the OSCE (almost three percentage points) than their classmates. Thus, arithmetically levelling differences between students not only balances performances, it even leaves the (professionally) experienced better off. Adding Abitur grade and students’ age as covariates only slightly increases R^2^, indicating that their impact on total variation in OSCE results is less pronounced, compared to MC exams.

In the assessment of medical skills (columns (4) to (6) in Table 4), participants’ gender does not play a role, and the influence of nationality and school background is less pronounced than in the overall OSCE result. Only students from the special quotas differ significantly from their peers in columns (4) and (5). Controlling for age and Abitur (model (6)) resolves this difference, and the estimate for waiting list students’ score is now 6 percentage points above their NC-quota classmates. The overall negative impact of age and Abitur grade on the OSCE outcome does not differ from column (3). Compared to the total OSCE score the R^2^ in columns (4)-(6) is markedly lower. This can be explained by the overall low variation in the two skills-stations, where most students perform very well.

The results for the OSCE part Communication (columns (7)-(9) in Table 4) resemble the outcomes for the whole examination. We find a strong positive impact of the variable German, which is no surprise, since an anamnesis and an empathetic consultation with a standardised patient requires a profound conduct of the language and technical terms. Females perform slightly better than males, while, in column (9), neither age nor the Abitur grade play a role in the assessment of communication. However, controlling for those factors dissolves statistically significant differences between NC, selection, and waiting list students.

#### Non-linear regression analysis

**Fig. 2:**
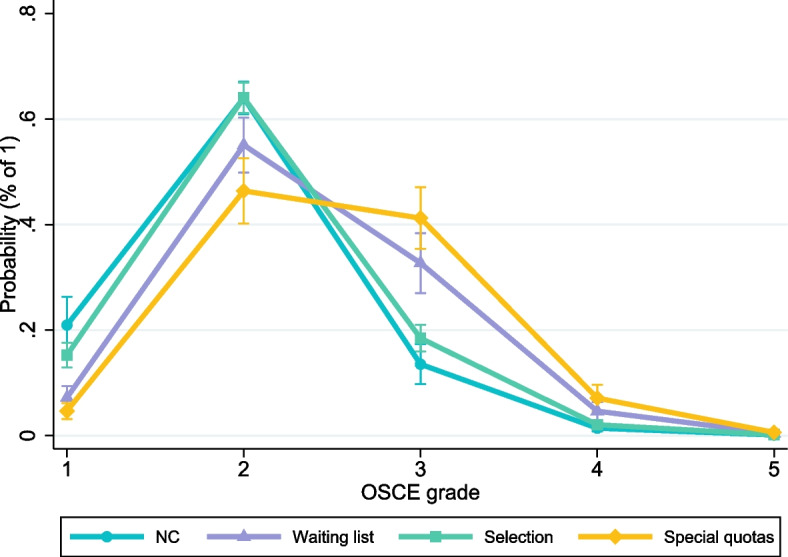
Predictive probabilities to reach different OSCE grades. Note: Marginal effects per selection quota and grade outcome, based on an ordered logistic regression (Table A in the Online Appendix). Calculations of the marginal effects are at the mean values of all control variables included in the regression model. 95%-confidence intervals are indicated

To illustrate how selection quotas and sociodemographic characteristics affect the probabilities to reach different grades in the OSCE, we use Fig. 2 as the graphical representation of an ordered logistic regression (see Table A in the Online Appendix). NC and selection students have a significantly higher chance (the confidence intervals do not overlap) to achieve a ‘very good’ (1) or ‘good’ (2) grade in the OSCE, compared to the waiting list group and special quotas. The chances for the grade ‘3’ (C) are significantly higher for the latter two groups. Thus, while controlling for Abitur grade, age, and other factors explain performance gaps, NC and selection students still can be expected to be more successful in the OSCE (overall). Figure 2 further shows that a good grade is the most common outcome for all groups, although for the special quotas an ‘average’ grade is almost as likely. Just passing the OSCE with the grade ‘adequate’ (4) or even failing the exam (the grade ‘5’) is highly unlikely, and the predictive probabilities do not differ across the four quotas.

#### OSCE performance and MC exams

**Fig. 3:**
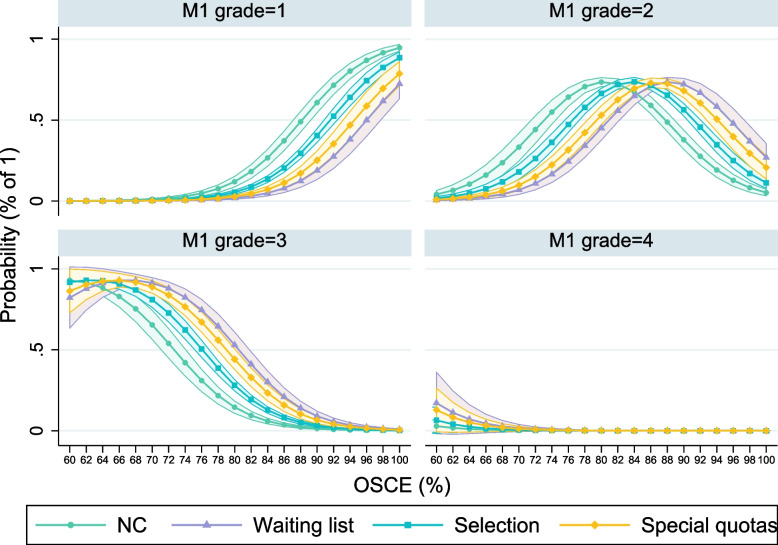
Relations between OSCE score and M1 grades, per selection quota. Note: Calculations at possible outcomes in the OSCE (scores in %). The depicted partial effects are from model (2) in Table B in the Online Appendix. Calculations of the marginal effects are at the mean values of all control variables included in the regression model. 95% confidence intervals are indicated

Figure 3 shows the association between OSCE performance and overall M1 grade of those students who had passed all courses in the two years of study by the time they participated in the OSCE. The calculations in Fig. 3 rely on an ordered logistic regression (with the M1 grade as the ordinal dependent variable) and depict the probability of reaching a specific M1-grade in relation to the total percentage scored in the OSCE. The full regression output can be found in the Online Appendix (Table B).

Students without an excellent overall OSCE score almost never (regardless of the admission quota) reach a ‘very good’ (1) final grade in M1. However, for solid good (i.e. more than 85% in the OSCE) or very good performances, chances for an excellent M1 increase over-proportionately. With a near-perfect OSCE, NC students have an at least 90% probability to achieve the best possible grade in M1 as well. Students from the selection group have a significantly smaller likelihood of a very good M1, and waiting list as well as special quotas perform significantly weaker than their classmates who passed the MHH’s selection process.

A ‘good’ grade is the most common in M1, and NC as well as selection students reach it significantly more often with a ‘satisfactory’ performance in the OSCE, compared to the waiting list and special quotas. Regardless of the selection procedure, the likelihood for the grade ‘3’ (C) in M1 decreases strongly with a better OSCE. Yet, except for low (60%-70%) and very high scores (> 90%), students from the waiting list (and the special quotas) have a significantly higher probability to obtain M1 with a ‘satisfactory’ degree. The worst passing grade in M1 as well as in the OSCE is very rare, and consequentially we do not find any statistically significant differences across admission quotas.

In Figure A (in the Online Appendix) we repeat the ordered logistic regression with the scores in the OSCE-subparts as dependent variables. Interestingly, the association between an excellent OSCE and a very good M1 is distinctly stronger for the parts internal medicine and neurology, compared with medical skills and communication. Figure A also reinforces the assessment that performance differences between NC (and selection) students and their waiting list classmates are larger in the former parts of the OSCE. Irrespective of the station and the results, the predicted chances to reach different M1 grades are nearly congruent for the waiting list and the special quotas groups.

## Discussion

The summative OSCE at MHH represents one of the essential final exams at the end of the first two academic years. Each of the nine OSCE stations demand different knowledge, skills and abilities. Medical students are aware of its importance and therefore prepare themselves accordingly. In general, competency-oriented and summative examinations allow for a differentiation between students with good and bad performances, but also motivate and support students’ outcome-oriented learning strategies (“*assessment for learning*”) [25, 26].

Reflecting on the research objectives we set out in the Introduction of our paper, we have shown that:

- 1) Waiting list students with working experience in adjacent health-care professions outperformed their classmates who entered university right after graduating from High School with excellent Abitur grades in the demonstration of practical, medical skills. Here, waiting list students can arguably take advantage of their existing professional experiences, giving them a slight edge in the application of medical craftsmanship. Students from the NC and selection quota performed better in the assessment of communicative skills. Because vocational training in Germany is less academic than in most other countries and trainees will have interacted with patients in the predominant on-the-job training (compared to the time spent in classes), this result does not entirely meet our expectation.
- 2) We confirm and reinforce the importance of cognitive abilities, in particular Abitur grades, as a predictor for study success in medical school. This applies to the competence-based parts of the OSCE, to a lesser degree also to the assessment of medical and communicative skills, and to M1-results overall.
- 3) We additionally find that success in the OSCE depends on performances in written and oral tests preceding the clinical examination: a high probability of a ‘very good’ or ‘good’ M1 is interrelated with strong performances in the OSCE (see Fig. 3). Those students who score less than 80% in the clinical examination seldom reach a good or very good (equivalent to an A or B) M1 grade. We think this reflects that students with a higher affinity to learning and rigorous preparation will be more successful in both MC exams and the OSCE. NC and selection students are more likely to reach excellent exam results. This is another indication to the significance of cognitive abilities for the ability to learn and thus for study success. An exception are the skills-stations, where practical experiences are more important, and waiting list students the most successful group.

The module ‘Diagnostic methods’ with the OSCE is a connecting element between the predominantly preclinical and the more practice-oriented parts of the medical curriculum. It appears to be a good assessment of all competences and skills taught in the first two years of study, and might have even predictive validity for study success in later years. Our findings are consistent with previous studies and emphasise the importance of a good knowledge base for the OSCE participation [26–28].

Previous evidence from MHH showed that students from the waiting list eventually slightly narrow the gap to NC and selection students once clinical elements become more frequent in the curricula, although performance differences remain pronounced and dropouts among waiting list students significantly more frequent compared to NC and selection quotas [29]. Yet, also the vast majority (more than 80%) of waiting list students graduate, which raises questions if and to what extent vocational training should be incorporated into student selection. We argue that there are good reasons besides existing practical skills and high intrinsic motivation: from internal polls at MHH, we know that waiting list students less frequently pursue a doctoral project. A direct transition into professional life (in particular if they tend to primary care) could address the looming doctors’ shortage described at the outset of this paper. Unfortunately, career choices of graduates – separated by selection quota or vocational training – were not observable to us. With respect to e.g. educational, occupational and family background, but also regarding life experiences or disruptions, waiting list (and special quotas) students are a more diverse group, compared to e.g. NC students. Thus, including criteria such as vocational training facilitates access to university for groups that are under-represented in ‘classic’ selection procedures, and they adhere to the political efforts to align curricula to competences required in medical practice.

Given the indisputable importance of cognitive abilities for study success in medical school, we emphasize that vocational training should be utilized as one complementary, but not as the decisive selection criterion. In this vein, the waiting list arrangement – with the time spent on the list (dependent on the Abitur grade) as the sole eligibility criterion – was of course far from being ideal. The fact that applicants were willing to wait often six or more years and used the latency to work in health care, however, at the very least signals both determination and high intrinsic motivation to pursue a career as a physician.

Since 2020, German medical schools have replaced the waiting list arrangement with a 10%-quota for the professionally experienced. At MHH, applicants in this quota are ranked and admitted based on their performance in a study ability test (the Abitur grade is not considered), thus combining vocational training and cognitive abilities. After 2019, the MHH has also halted its selection interviews to fill 60% of places. For 20% of these spots, applicants can boost their scores from a study ability test and their Abitur grade if they can attest vocational training in a relevant health-care profession.

A limitation of our study is the still constrained set of covariates available to us. Students’ personalities, experiences, motivations, learning strategies and views determine and influence academic success, but were unobservable to us, giving potential to a slight omitted variable bias. While we know that (almost) all waiting list students and none of the NC-group have received vocational training, we have little information for the selection group and special quotas. From internal polls and surveys (not for our sample), we approximate that no more than 10%-15% of students in these groups have started vocational training. Because latency between finishing school and enrolment in these groups rarely exceeds more than a year, we are, however, confident that students have not finished vocational training and thus have had no work experience before entering medical school. Limited availability of variables is common to all observational studies on the links between student selection and study success. In line with the literature, we report robust correlations and evaluate predictive validity, but cannot deduct causal inferences from our study. We were able to include extensive data on six full cohorts and could exploit detailed exam results. However, our analysis was limited to only one medical school. Because contents and timing of the OSCE are specific to the MHH and its curriculum, we cannot generalize our results to other sites. Greater external validity could be achieved in future research with a multi-centric study.

Since training students to become ‘good doctors’ is the ultimate objective of medical education and implies much more than success in exams, we see the need for future research that links student selection and academic success with graduates’ career choices and job performances. This should ideally include research on how to evaluate what makes doctors ‘good’ (and which traits this term should encompass), besides academic performances.

## Supplementary Information

**Additional file 1: Table A.** Student selection and osce performance; ordered logit regressions. **Table B.** Osce performances and m1-grades; ordered logit regressions. **Figure A.** Relations between OSCE scores and M1 grades.

## Data Availability

The data analysed during this study is not publicly available due to its proprietary nature and privacy concerning administrative data used for this article. Data are available from the corresponding author upon reasonable request.

## Abbreviations

GPA: Grade point average
M1 (M1-equivalence): First part of the German national medical licensing exam
MC: Multiple choice
MHH: Hannover Medical School
NC: Numerus clausus
NKLM: Nationaler Kompetenzbasierter Lernzielkatalog Medizin (National competency-based learning objectives catalogue medicine)
OSCE: Objective Structured Clinical Examination
UK: United Kingdom
US: United States
