# Supplementary material for: Clinical experience can compensate for inferior academic achievements in an undergraduate objective structured clinical examination
Source: BMC Med Educ. 2023 Mar 16;23:167. doi: 10.1186/s12909-023-04082-x (PMC10022153; doi:10.1186/s12909-023-04082-x)
Supplement: Supplementary file 1 — Additional file 1: Table A. Student selection and osce performance; ordered logit regressions. Table B. Osce performances and m1-grades; ordered logit regressions. Figure A. Relations between OSCE scores and M1 grades. [file 12909_2023_4082_MOESM1_ESM.docx]

Clinical experience can compensate for inferior academic achievements in an undergraduate Objective Structured Clinical Examination

**Online Supplementary Material**

**Table A:** STUDENT SELECTION AND OSCE PERFORMANCE; ORDERED LOGIT REGRESSIONS

|  |  | **Reference group: NC** | | |  |  |  |  |  |
| --- | --- | --- | --- | --- | --- | --- | --- | --- | --- |
|  | Dependent variable | Waiting list | Selection | Special quotas | Female | German | Gymnasium | Age | Abitur grade |
| (1) | OSCE grade | 1.089*** | 0.340* | 1.104*** | -0.327*** | -1.329*** | -0.859*** |  |  |
|  |  | (0.220) | (0.174) | (0.236) | (0.119) | (0.251) | (0.162) |  |  |
| (2) | OSCE grade | -0.863** | 0.059 | 0.365 | -0.321*** | -1.711*** | -0.783*** | 0.072*** | 0.812*** |
|  |  | (0.436) | (0.190) | (0.282) | (0.122) | (0.268) | (0.172) | (0.025) | (0.221) |

*Notes:* Logistic regressions with the OSCE-grade as the ordered dependent variable. A graphical representation of marginal effects from specification (2) can be found in Figure 2 in the main text. Robust standard errors are in parentheses. *N* = 1,283 in models (1) and (2); Pseudo-*R*^2^: (1) 0.085, (2) 0.097; cohort fixed effects are included in both specifications. *: *p*<0.1; **: *p*<0.05; ***: *p*<0.01

**Table B:** OSCE PERFORMANCES AND M1-GRADES; ORDERED LOGIT REGRESSIONS

|  | (1) | (2) | (3) | (4) | (5) | (6) |
| --- | --- | --- | --- | --- | --- | --- |
| *OSCE part:* | *Total* | *Internal medicine* | *Neurology* | *Radiology* | *Medical skills* | *Commu-nication* |
|  |  |  |  |  |  |  |
| **Percent scored in OSCE-part…** | -0.252*** | -0.090*** | -0.059*** | -0.061*** | -0.067*** | -0.093*** |
|  | (0.015) | (0.008) | (0.005) | (0.006) | (0.007) | (0.009) |
|  |  |  |  |  |  |  |
| **Reference: NC** |  |  |  |  |  |  |
| Waiting list | -0.065 | -0.084 | -0.230 | -0.163 | -0.020 | -0.605 |
|  | (0.461) | (0.471) | (0.460) | (0.511) | (0.467) | (0.462) |
| Selection | 0.567*** | 0.575*** | 0.622*** | 0.500** | 0.664*** | 0.578*** |
|  | (0.200) | (0.191) | (0.183) | (0.218) | (0.189) | (0.188) |
| Special quotas | 0.782*** | 0.997*** | 1.039*** | 0.619* | 0.964*** | 0.772*** |
|  | (0.295) | (0.279) | (0.283) | (0.323) | (0.292) | (0.282) |
|  |  |  |  |  |  |  |
| Female | 0.657*** | 0.397*** | 0.367*** | 0.455*** | 0.402*** | 0.459*** |
|  | (0.139) | (0.130) | (0.132) | (0.147) | (0.130) | (0.131) |
| German | -0.593** | -0.939*** | -1.338*** | -1.303*** | -1.526*** | -1.251*** |
|  | (0.273) | (0.260) | (0.267) | (0.332) | (0.269) | (0.263) |
| Gymnasium | -0.536*** | -0.762*** | -0.702*** | -0.584*** | -0.732*** | -0.641*** |
|  | (0.198) | (0.181) | (0.179) | (0.223) | (0.185) | (0.184) |
| Age | 0.067** | 0.046 | 0.072** | 0.075* | 0.074** | 0.092*** |
|  | (0.034) | (0.032) | (0.031) | (0.038) | (0.032) | (0.032) |
| Abitur grade | 0.939*** | 0.999*** | 1.074*** | 0.865*** | 1.055*** | 1.129*** |
|  | (0.249) | (0.243) | (0.250) | (0.276) | (0.245) | (0.248) |
|  |  |  |  |  |  |  |
| Cohort FE | YES | YES | YES | YES | YES | YES |
| *N* | 1,159 | 1,159 | 1,159 | 931 | 1,159 | 1,159 |
| Pseudo *R*^2^ | 0.280 | 0.176 | 0.171 | 0.177 | 0.149 | 0.165 |

*Notes:* Logistic regressions with the M1 grade as the ordinal dependent variable. Specifications (1)-(6) use the percent scored in the different parts of the OSCE as the main predictor. A graphical representation of marginal effects of specification (1) can be found in Figure 3 in the main text. Graphical representations of the other regression models are shown in Figure A of the Online Supplementary Material. Robust standard errors are in parentheses.

*: p<0.1; **: p<0.05; ***: p<0.01

Panel A: Internal medicine


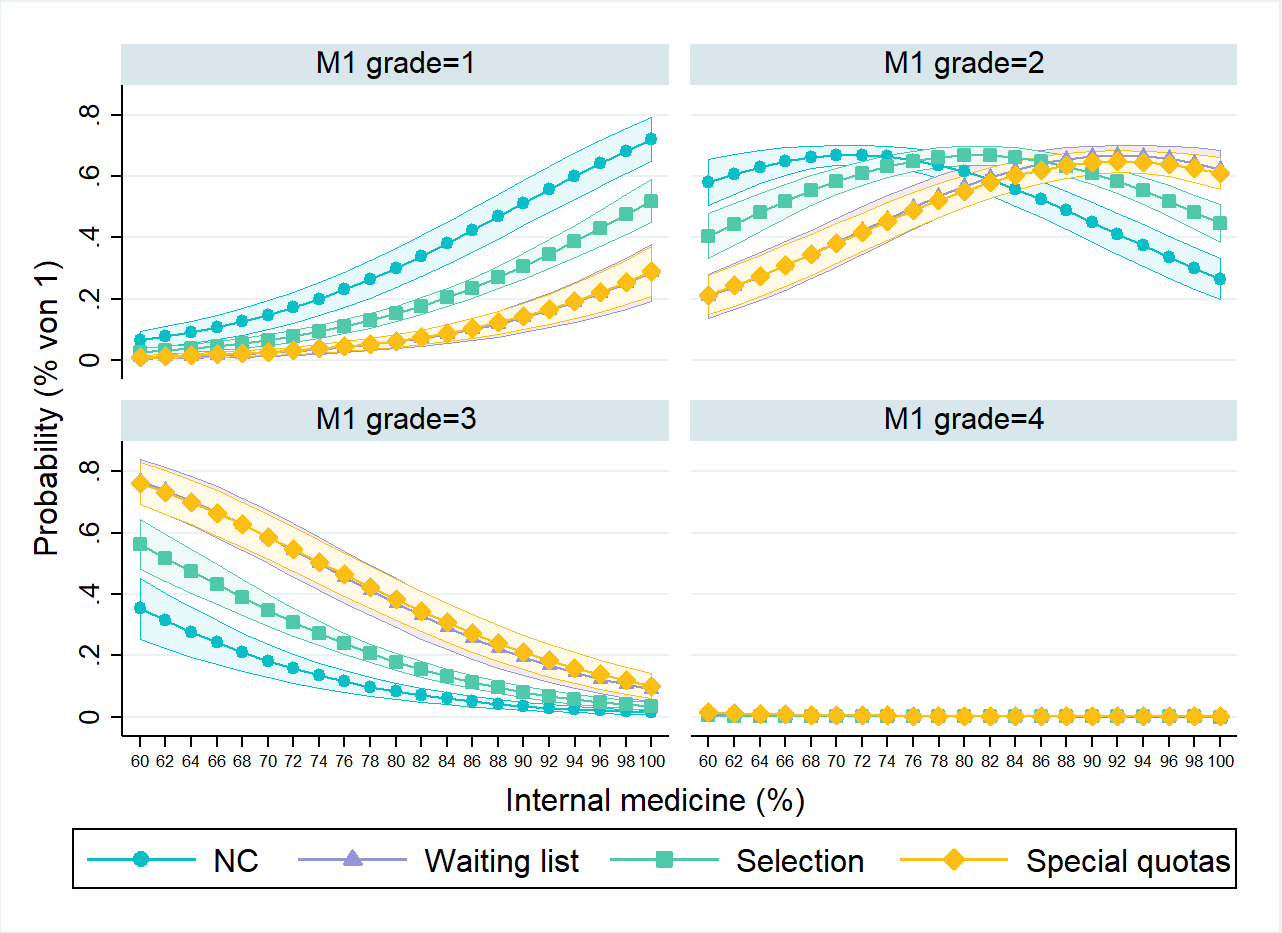


Panel B: Neurology


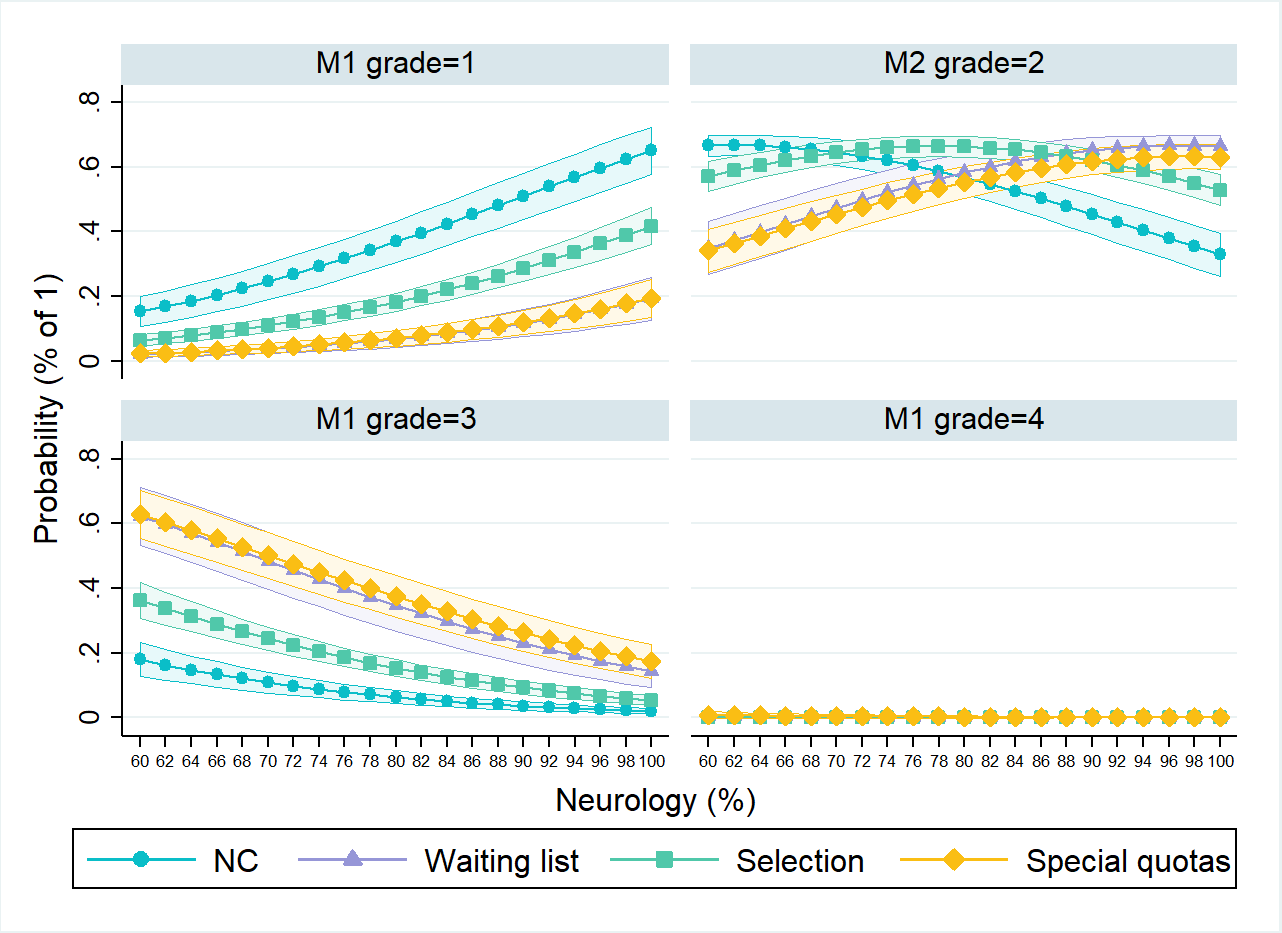


**Figure A:** Relations between OSCE scores in internal medicine (panel A), neurology (panel B) and M1 grades

*Notes: graphs by selection quota, 95% confidence-intervals are indicated. Graphs based on ordered logistic regressions and calculated at the mean of the sociodemographic variables (see Table 1)*

Panel C: Radiology


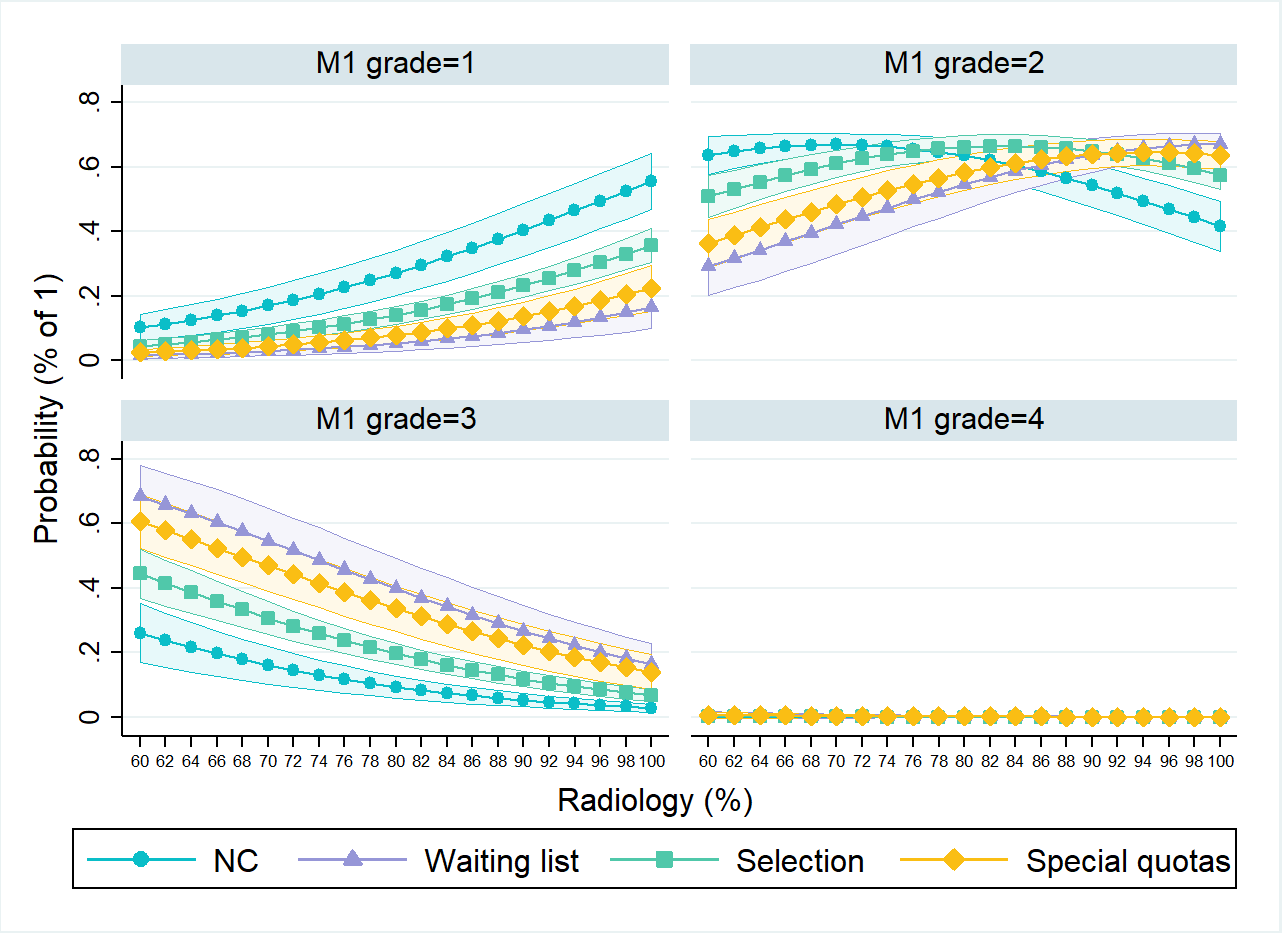


Panel D: Medical skills


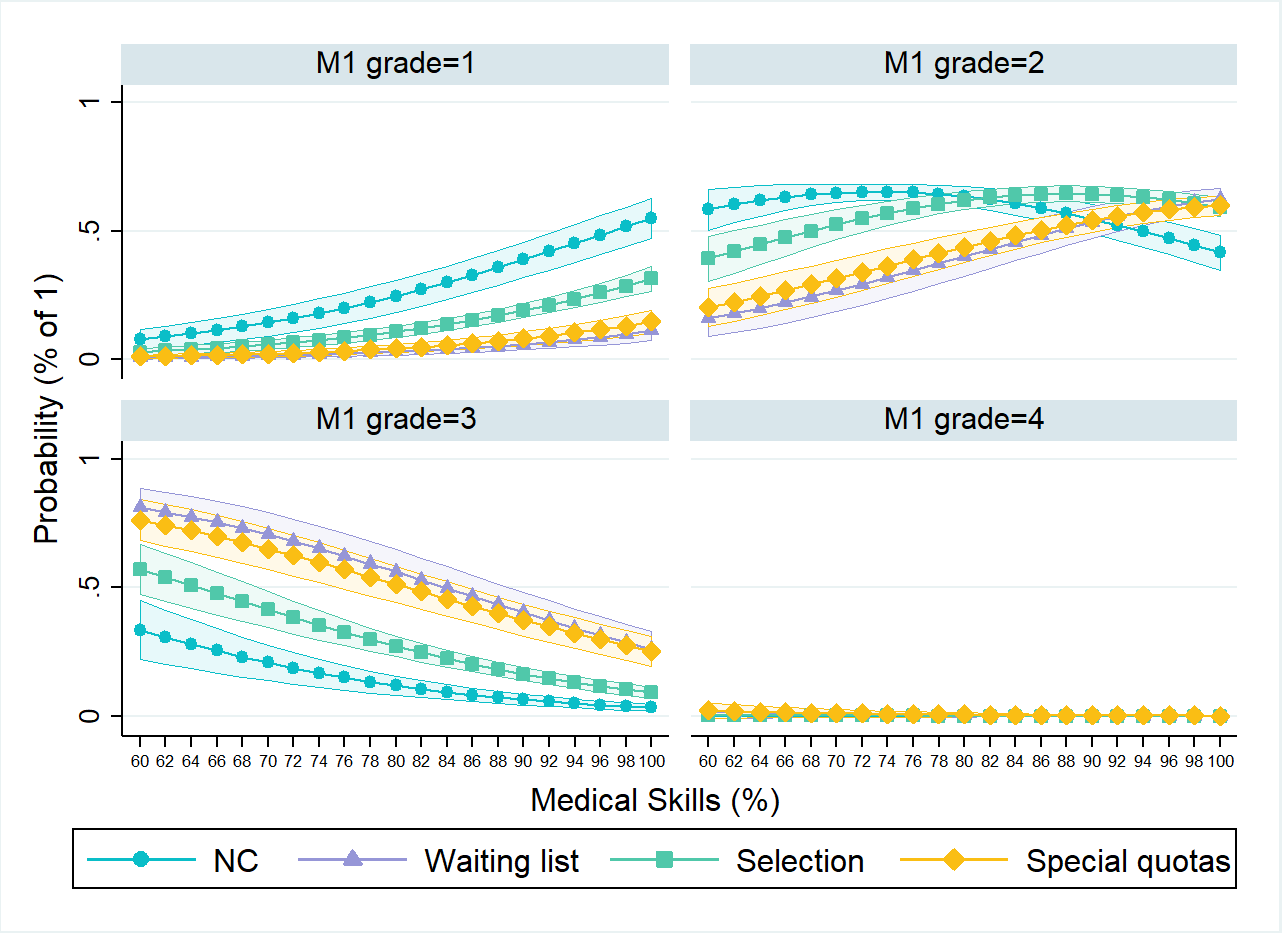


**Figure A (cont’d):** Relations between OSCE scores in radiology (panel C), medical skills (Panel D) and M1 grades

Panel E: Communication


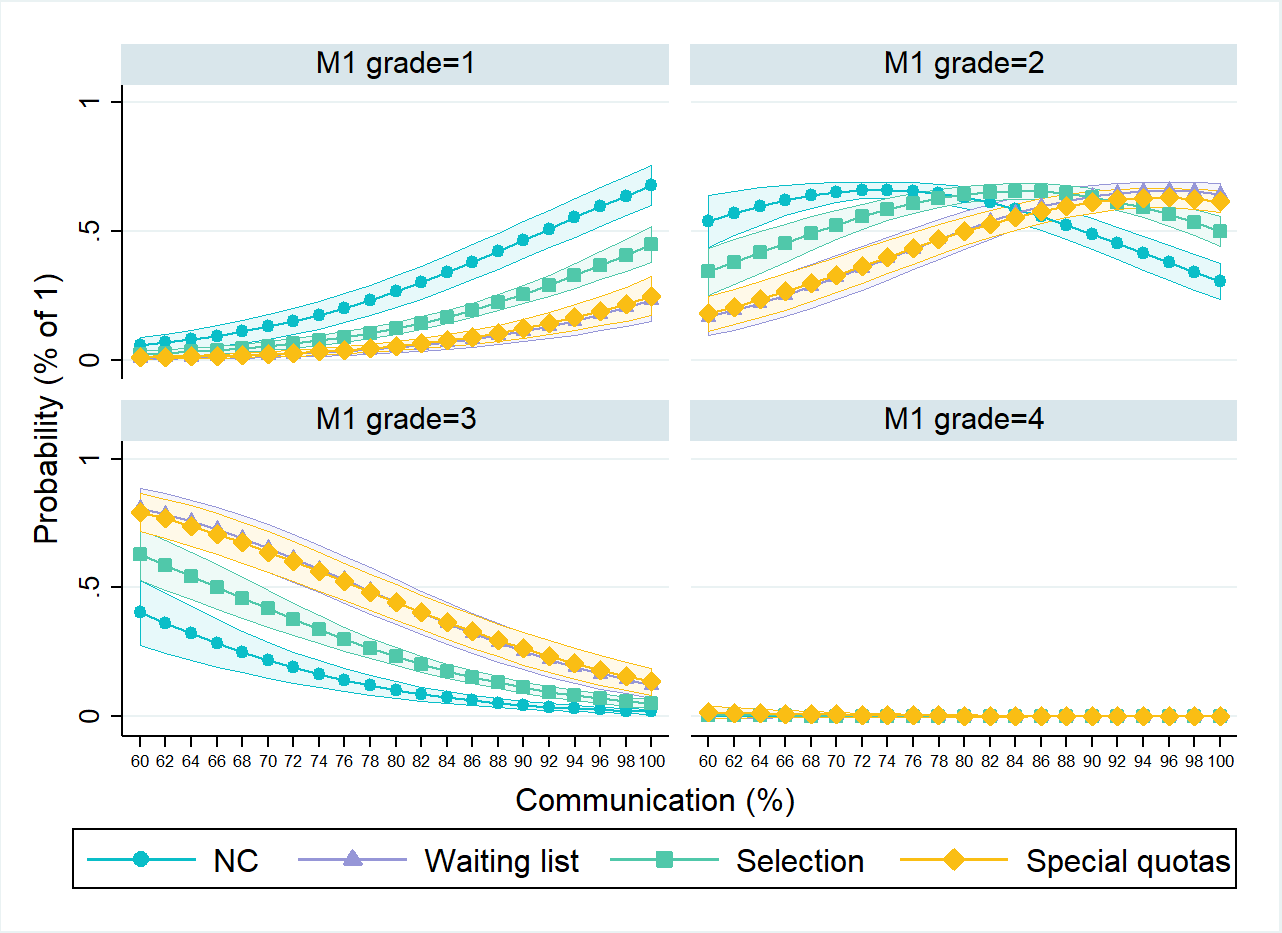


**Figure A (cont’d):** Relations between OSCE scores in doctor-patient interaction (panel E) and M1 grades
